# The Impact of Teaching Multiple Responses on Resurgence of Target Behavior and Persistence of Alternative Responding

**DOI:** 10.3390/bs15081014

**Published:** 2025-07-25

**Authors:** Brittany H. Loder-Lafferty, Amanda N. Zangrillo, Alexandra M. Cicero, Cynthia P. Livingston, Jessica P. Tran, Mark Connelly

**Affiliations:** 1 Munroe-Meyer Institute, University of Nebraska Medical Center, Omaha, NE 68106, USA; anzangrillo@cmh.edu (A.N.Z.); acicero@unmc.edu (A.M.C.); clivingston@unmc.edu (C.P.L.); jtran@unmc.edu (J.P.T.); 2 Division of Developmental and Behavioral Health, Children’s Mercy, Kansas City, MO 64108, USA

**Keywords:** autism, functional communication training, resurgence, treatment durability, communication preference

## Abstract

This study evaluated the effects of teaching multiple alternative responses on the resurgence of a target response and the persistence of an alternative response in an applied setting. Using a between-participants design, we examined how teaching multiple alternative responses impacted resurgence and persistence upon exposure to extinction. Additionally, we investigated the role of preference in response allocation and shifts in participant preference following extinction. Results indicated resurgence across both conditions, with no consistent difference in severity between single and multiple alternative response conditions. However, within-session analyses revealed greater persistence of the alternative response for participants taught multiple alternative responses, suggesting potential benefits for sustained engagement. Future researchers should continue to investigate the role of preference, as teaching order may have impacted findings. Despite mixed findings, this study provides valuable insights into clinical strategies for promoting alternative responding. While teaching multiple alternative responses may not prevent resurgence, it may enhance communication flexibility, particularly when certain responses become unavailable.

## 1. Introduction

Studies have demonstrated the efficacy of functional communication training (FCT) across many preparations of the procedure (see Ghaemmaghami et al., 2021 or Tiger et al., 2008 for a review). However, challenges (e.g., programmed treatment challenges and unplanned treatment interruptions) may arise during intervention despite well-intentioned consistent implementation. These challenges can result in the relapse or reemergence of challenging behavior and a decrease in allocation toward the appropriate alternative response (e.g., functional communication response; see Perrin et al., 2022 or Radhakrishnan et al., 2020 for a review). Relapse refers to the reemergence of previously eliminated target behavior (e.g., challenging behavior), and resurgence may particularly occur when an alternative response, such as a functional communication response, is not reinforced (i.e., placed on extinction; Perrin et al., 2022; Volkert et al., 2009). For example, schedule thinning, a deliberate treatment procedure aimed at decreasing the frequency of reinforcement for a communication response, has been associated with increased rates of resurgence during its application due to the worsening of reinforcement conditions (Briggs et al., 2018; Hagopian et al., 1998; Nuhu & Pence, 2022; Rooker et al., 2013). Similarly, accidental treatment interruptions, such as errors of omission (i.e., not providing reinforcement for the alternative response, short supply or unavailability of the reinforcer) may also lead to resurgence (Mitteer et al., 2018; Mitteer et al., 2021).

Researchers have employed a three-phase resurgence preparation to better understand the variables influencing both resurgence of the target response and persistence of the alternative response under worsening reinforcement conditions (Lattal & St. Peter Pipkin, 2009; Lattal et al., 2017; Sweeney & Shahan, 2015). In phase one, the researcher or clinician programs reinforcement for the target response (e.g., challenging behavior). During phase two, they shift reinforcement to an alternative response(s) (e.g., functional communication response) while placing the target response on extinction. Finally, in phase three, the researcher or clinician programs extinction for all responses (i.e., target and alternative). Resurgence is said to occur if the observed rate of target responding within phase three is at or above the rate of target responding in phase two (Lattal & St. Peter Pipkin, 2009; Lattal et al., 2017; Sweeney & Shahan, 2015).

Researchers have explored various strategies to mitigate resurgence while promoting engagement in the alternative response during worsening reinforcement conditions. Prior research has investigated strategies such as signaling the availability and unavailability of reinforcement, manipulating reinforcement schedules, and altering the value and effort of a response (see Kimball et al., 2023 for a discussion). Two additional strategies that have received emerging focus within the literature involve programming multiple alternative responses (e.g., Lambert et al., 2017) and incorporating preference for an alternative response (e.g., Ringdahl et al., 2018).

### 1.1. Programming Multiple Alternative Responses

Expanding the number of alternative responses may increase the value of and shift responding toward the alternative response and away from the target response (Greer & Shahan, 2019); however, recent research investigating this has reported mixed results. Lambert et al. (2015) investigated the effects of teaching multiple alternative responses on the resurgence of target responding during extinction for three adults with developmental disabilities. Using a three-phase resurgence preparation, the researchers compared conditions in which participants were taught either single (control) or multiple (test) alternative analog responses. Participants in the test condition experienced increased alternative responding and reduced resurgence of the target response during phase three, suggesting potential benefits to teaching multiple alternative responses to mitigate resurgence.

Lambert et al. (2017) extended these translational findings to an applied setting with two individuals engaging in challenging behavior. While participants showed higher alternative responding in the test condition, resurgence effects varied. Several limitations could contribute to the inability to replicate the results of Lambert et al. (2015). Specifically, Lambert et al. (2017) varied the type of alternative responses, including some that required physical stimuli (e.g., switch, card exchange) and others that did not (e.g., sign language), with the potential of physical stimuli visually prompting or signaling availability of reinforcement for some responses. Additionally, for one participant, obtained reinforcements for the alternative responses during phase two were not equated across the test and control conditions, potentially impacting the relative levels of resurgence observed during phase three (Podlesnik & Shahan, 2009, 2010; see Shahan & Sweeney, 2011, for a discussion). Given contradicting findings, additional research was warranted.

Diaz-Salvat et al. (2020) replicated findings from Lambert et al. (2015) in a translational setting, while also extending the research to evaluate the impact of mere availability of alternative responses, as opposed to programmed teaching and reinforcement. The authors concluded that increased response options, independent of teaching and reinforcement, could mitigate resurgence and increase responding toward the alternative response. The authors, however, expressed caution in the clinical utility of the results given the physical similarity between target and alternative response options (i.e., circles on a screen) and inability to rule out response generalization and its impact on responding. Despite limited clinical utility, these findings highlighted the importance of including control responses to assess for potential faulty stimulus control. Additionally, different response topographies should be considered to rule out response generalization.

All aforementioned studies employed the use of a within-subject design, which may introduce additional confounding variables as questions remain regarding the replicability of resurgence effects across repeated exposure to extinction (Redner et al., 2022). Specifically, some research has demonstrated that multiple exposures to extinction may increase resurgence (e.g., Kimball et al., 2018; Marsteller & St. Peter, 2014), while others have reported a decrease in observed resurgence (e.g., Kestner et al., 2018; Podlesnik et al., 2020) or no change (e.g., Wacker et al., 2013). Therefore, other designs that use alternatives to within-subject replication may be better suited to limit confounding variables and investigate the effects on the levels of resurgence. Fuhrman et al. (2021) expanded the literature by employing a between-participants group design with neurotypical adults, addressing the limitations of using a within-subject design. Fuhrman et al. found increased alternative responding in the test condition relative to the control but no difference in the observed levels of resurgence. However, the authors used similar response options for both target and alternative responses, limiting the ability to conclude if the findings were, again, the result of response generalization or teaching multiple alternative responses.

Taken together, these studies suggest that teaching multiple alternative responses may promote persistence of appropriate alternative responses, but the effects on levels of resurgence have produced heterogenous findings. Given the conflicting findings and methodological differences (e.g., reinforcement rates, stimulus variability, lack of control response, design) in previous research, this highlights the need for further investigation, particularly into the mechanisms driving resurgence and the design features that best control for confounding variables.

### 1.2. Incorporating Preference for an Alternative Response

There has also been increasing popularity of investigating the impact that preference for an alternative response has on the resurgence of a target response and persistence of the alternative response. Several studies have reported that individuals with developmental disabilities may demonstrate preference for a specific modality of communication incorporated during treatment (Kunnavatana et al., 2018; Livingston et al., 2025; Ringdahl et al., 2024, 2016; Winborn-Kemmerer et al., 2009), and incorporating these more preferred communication modalities may bias responding toward communication in place of the target response during contact with extinction (Ringdahl et al., 2018). Additionally, incorporating a preferred communication modality may also increase opportunities for the involvement of the client in treatment and increase the social validity of intervention procedures (Ringdahl et al., 2024). Therefore, additional research investigating the effect of preference on the persistence of the alternative response and resurgence of the target response may reveal important clinical implications.

In this current evaluation, we built on past literature, prioritizing gaps in previous research that remain unanswered. Our research question was: How does teaching multiple topographically dissimilar alternative responses, compared to a single alternative response, affect the resurgence of target behavior and the persistence of alternative responding in individuals exhibiting challenging behavior in applied settings? The purpose of this study was to compare the effect of teaching a single alternative response (control condition) versus teaching multiple topographically dissimilar alternative responses (test condition) on the resurgence of target and persistence of alternative responding in an applied setting for individuals engaging in challenging behavior. To address prior gaps and limitations, we specifically incorporated different physical stimuli for the alternative responses to ensure consistency of physical stimuli, programmed a control response and different response topographies for the alternative and target responses to control for response generalization, implemented procedures to assist in balancing reinforcers for the alternative response, and used a between-groups design to control for multiple exposures to extinction. For participants taught multiple alternative responses, we then evaluated the relative preference for the available alternative responses and the potential relationship this may have had on the persistence of responding during contact with extinction. Finally, we investigated changes in preference for alternative responses following contact with extinction.

## 2. Materials and Methods

### 2.1. Participants

Participants met inclusion criteria if they (a) were referred to an outpatient program for the assessment and treatment of severe challenging behavior and (b) engaged in challenging behavior hypothesized to be maintained by social negative and/or social positive reinforcement (hypothesized by the caregiver interview and descriptive observation). We excluded participants from enrollment if they engaged in challenging behavior maintained by only automatic reinforcement (determined by functional analysis (FA)) and/or participant FA results were determined as undifferentiated by the researcher and clinical team. Fourteen participants met inclusion criteria, and six participants were disenrolled from the study. Three participants were disenrolled due to undifferentiated FA results, one due to inability to evoke responding during phase one of the evaluation, and two participants were disenrolled due to fleeting motivating operations impeding ability to acquire a functional communication response. The eight remaining participants completed all portions of the study. Table 1 depicts participant characteristics and demographics (i.e., age, sex assigned at birth, race, diagnosis, and spoken language) for the participants who completed all portions of the study.

### 2.2. Setting and Materials

We conducted all sessions at an outpatient clinic, in padded session rooms that measured approximately 3.7 m by 3 m. The rooms included padded walls and floors and contained a one-way mirror for unobtrusive observation by data collectors. Session materials included tables and chairs; communication materials used throughout FCT (e.g., voice-generating button, iPad, laminated communication cards); a control card; high-, moderate-, and low-preferred items identified via a preference assessment; and academic materials when appropriate.

### 2.3. Response Measurement and Definition

Trained data collectors used a laptop computer with specialized data collection software (DataPal 1.0; Bullock et al., 2017) to collect real-time data across sessions. Data collectors recorded information on the target, alternative, and control card responses.

Target responses were individualized for each participant (Table 2). We defined aggression as the participant’s body parts making forceful physical contact with another person’s body, pushing, pinching, or scratching; disruption as forcefully hitting and/or throwing items to the ground, wall, or tables and tearing items; and self-injury as forcefully hitting participants’ own face, head, or other body parts, self-choking, self-biting, or hair pulling. For Cooper, we defined vocal protests as a statement of refusal (i.e., “No”, “I don’t want to”). For Deavon, we defined elopement as any completed, attempted, or blocked instance of leaving a supervised area. This included touching the door handle or opening the door while inside the session room, moving out of an already opened session-room doorway, moving from a supervised room or area without permission, or moving more than 5 feet away from the therapist.

During all phases of the evaluation, we collected the frequency of target responding and total duration of each session. We then calculated a rate of target responding by dividing the frequency of target responses by the total duration of session. During phase two of the evaluation, we also collected the frequency of trials with and without target responding. We then converted this to a percentage of trials with target responding by dividing the number of trials with target responding by the total number of trials in a session and multiplying by 100.

Alternative response(s) taught during this study included externally aided functional communication response modalities. These modalities included either one or multiple of the following: (a) card exchange, (b) card touch, (c) speech-generating button, or (d) speech-generating device. We scored a card exchange if the participant placed the programmed communication card into the therapists hand, card touch if the participant touched the programmed communication card for 2–4 s and removed their hand/finger, speech-generating button press if the participant pressed the programmed button with enough force to activate the speech output, and speech-generating device if the participant pressed the application icon with enough force to activate the speech output. We scored an alternative response as independent if the participant independently touched or exchanged the programmed alternative response, activated a speech-generating button, or touched the programmed speech icon on the alternative response in the presence of the establishing operation (i.e., presence of instruction) without engaging in the target response. We scored an alternative response as prompted if the participant emitted the programmed alternative response immediately following a therapist’s prompt. For participants in the test condition, we scored a blocked alternative response if the therapist blocked engagement toward a non-targeted alternative response in the presence of the establishing operation.

During phases two and three of the study, data collectors scored the frequency of independent, prompted, and blocked alternative responses. Data collectors then calculated a rate of alternative responses by summing the frequency of independent and prompted alternative responses and dividing it by the total session duration. During phase two sessions, data collectors also calculated a percentage of independent alternative responses by dividing the frequency of independent alternative responses by the sum of independent and prompted alternative responses. For participants in the test condition, data collectors also scored the frequency of blocked alternative responses and obtained a rate by summing blocked alternative responses and dividing it by total session duration.

We scored engagement with the control card if the participant touched the control card for 2–4 s and removed their hand/finger, or exchanged the control card with a therapist, during any sessions. Data collectors obtained a rate of control card responses by dividing the frequency of control card responses by session duration.

### 2.4. Data Analysis

We evaluated the presence of resurgence by comparing the rate of target responding in phase three to the rate of target responding in the last three sessions of phase two. We also conducted additional analyses with the collected data. First, if target responding occurred in the first session of phase three, we calculated the difference between the aggregated rate of target responding in the last three sessions of phase two to the first session in phase three with any obtained values above 0.0 indicating resurgence. We also calculated a proportion of baseline for each session of phase three for each participant by calculating the average rate of target responding during the last three sessions of phase one and dividing the rate of target responding during each session of phase three by the phase one average, with values above 1.0 indicating increased responding relative to baseline and values below 1.0 indicating decreased responding relative to baseline. In addition to a proportion of baseline, we calculated a proportion of alternative responding for the first session of phase three. This was calculated by taking the rate of alternative responding during the first session of phase three and dividing it by the sum of alternative and target responding in the first session of phase three, with values above 1.0 indicating increased alternative responding relative to target responding and values below 1.0 indicating decreased alternative responding relative to target responding. Lastly, we analyzed the latency to the first alternative and target response during the first session of phase three by reporting the time it took each participant to engage in an alternative and target response following the presentation of the establishing operation.

For the test and control condition, we summed the obtained participant values for the difference in target responding and divided the value by the total number of participants generating an average difference in target responding. This was repeated for proportion of baseline, proportion of alternative responding, and latency to first alternative and target response. To evaluate whether differences between conditions were statistically significant (i.e., beyond what might be expected by chance), we used the Mann–Whitney U test. This non-parametric test evaluates whether the distribution of ranks differs between two conditions on a given behavioral measure, such as response latency or rate, indicating whether values in one group tend to be higher or lower than in the other. This method was selected due to the small sample size and potential non-normality of the data. In recognition of the limited statistical power inherent in small samples, we also calculated Cliff’s Delta, a rank-based non-parametric effect size index that estimates the practical significance of differences observed between groups and is not inherently dependent on sample size. Cliff’s Delta values of approximately 0.11, 0.28, and 0.43 are typically interpreted as small, moderate, and large effects, respectively.

### 2.5. Interobserver Agreement and Procedural Integrity

A second trained observer independently collected data on target, alternative, and control card responses for all participants for at least 41% (range: 41–67%) of sessions. To calculate interobserver agreement, we divided sessions into 10 s intervals. The total number of intervals for which both observers agreed on the frequency of responses was divided by the total number of intervals and multiplied by 100 to yield a percentage. Average total agreement across all participants was 96% (range: 85–100%).

We calculated procedural integrity for all participants for at least 33% (range: 33–73%) of sessions across each phase of the evaluation. We divided the number of correct steps by the number of total steps and converted to a percentage to yield a percentage of procedural integrity. For all phases of the study, average total procedural integrity was 99.7% (range: 99.3–100%).

### 2.6. Procedures

#### 2.6.1. Communication Resource Interview

The experimenters completed a communication resource interview (informed by the work of Gibson et al., 2020; see Supplementary Materials for interview) with caregivers to inform the selection of alternative response modalities used during this study. The interview included guided questions about the participants’ current and previously used modalities of communication. The experimenters then asked caregivers to rank order a list of four communication modalities (e.g., card touch, card exchange, speech-generating button, and speech-generating device) for their preference and for their perceived feasibility.

#### 2.6.2. Three-Phase Resurgence Preparation

All participants underwent the three-phase resurgence preparation. For test condition participants only, we assessed alternative response preference following the teaching of the alternative responses. We placed participants into matched dyads to allow for the balancing of obtained reinforcers for the alternative responses across participants in the test and control conditions (see Fuhrman et al., 2021 for an example). A single test participant was placed in a dyad with a control counterpart based on time of enrollment in the study. We programmed the first participant in each dyad to the test condition because we hypothesized participants would take longer to acquire multiple alternative responses when compared to a single alternative response. To balance obtained reinforcers for the alternative response(s) across participants within a dyad, we yoked the number of obtained reinforcers for the alternative response (within 10% range) for the participant in the control condition to that of the test condition (including communication preference assessment procedures (i.e., mixed trial and communication modality multiple stimulus without replacement [MSWO] preference assessment)).

Phase One. Phase one sessions were identical to the FA test condition that evoked the highest level of responding with the addition of a black control card present during sessions. Phase one sessions were 5 min (10 min for Kingston) and were conducted for a minimum of three sessions or until stability in responding was achieved via visual inspection, evidenced by either stable responding in a level trend or an increasing trend.
Phase Two. Phase two included teaching the alternative response(s) and sessions were trial based, consisting of 10 trials per session. The researchers used the information obtained from the communication resource interview to inform selection of the alternative response(s) included within the evaluation. Specifically, we weighted each ranking of caregiver preference and perceived feasibility for the communication modalities. The most feasible and preferred ranked modalities were assigned a score of 1, the second most feasible and preferred modalities ranked were scored a 2, and so on. We then summed the score for preference and feasibility, and overall response modalities were ranked in order of lowest to highest scored.

Test Condition. In the test condition, therapists taught participants three different alternative responses (Alt 1, Alt 2, Alt 3) in a sequential order (Lambert et al., 2015). The first three lowest scored response modalities from the communication resource interview were selected for teaching. Following the mastery of the alternative responses (see criteria below), participants within the test condition also participated in a preference assessment to evaluate relative preference for the alternative responses (i.e., mixed trial and MSWO communication preference assessment).

Alt 1. Prior to the beginning of the session, the therapist placed Alt 1 and the control card equidistant in front of the participant and provided the participant with the functional reinforcer (e.g., attention, tangible item, escape) for 1 min. The trial began once the therapist presented the establishing operation (i.e., removal of functional reinforcer) while prompting the participant to engage in Alt 1. The therapist provided the functional reinforcer for 30 s contingent on Alt 1. This continued for 10 total trials. The position of the alternative response and control card were alternated between trials. All sessions included an embedded progressive prompt delay (e.g., 0 s, 5 s, 10 s) at the controlling prompt level sufficient to evoke the correct response in the presence of the establishing operation (Charlop et al., 1985). The prompt delay was increased following two consecutive sessions with target responding occurring for 20% or fewer trials. The therapist programmed extinction for target and control card responses. Therapists also implemented a differential reinforcement of other behavior procedure, withholding reinforcer delivery if an alternative response co-occurred within 3–5 s of the target or control card response. Therefore, participants did not receive reinforcement for engagement in the control card or target response. Sessions continued until the participant met mastery criteria (i.e., completed two consecutive sessions with 80% of trials with independent targeted alternative responses and 20% or fewer trials with target responses). Following mastery, sessions for Alt 1 were terminated and the therapist initiated sessions for Alt 2.

Alt 2. Alt 2 sessions were identical to Alt 1. However, in Alt 2 sessions, Alt 1, Alt 2, and the control card were all present and placed in a straight line equidistant in front of the participant. If the participant attempted to engage in Alt 1 during the trial, the therapist blocked the attempt and immediately prompted Alt 2. If the participant was successful at engaging in Alt 1, the therapist placed the response on extinction and immediately prompted Alt 2.

Alt 3. Alt 3 sessions were identical to Alt 1 and Alt 2 sessions. However, in Alt 3 sessions, Alt 1, Alt 2, Alt 3, and the control card were all present and placed in a straight line equidistant in front of the participant. If the participant attempted to engage in Alt 1 or Alt 2 during the trial, the therapist blocked the attempt and immediately prompted Alt 3. If the participant was successful at engaging in Alt 1 or Alt 2, the therapist placed the response on extinction and immediately prompted Alt 3.

Control Condition. Participants assigned to the control condition were taught a single alternative response. The experimenter selected and taught the lowest scored response modality from the communication resource interview. Session procedures were identical to Alt 1 sessions detailed above. Following mastery of the alternative response, the participant remained in phase two sessions for the designated number of sessions (i.e., within 10% of sessions of the test condition participant) to equate obtained reinforcement for the alternative responses between the test and control condition participants.

Phase Three. Following completion of phase two (and preference procedures for test participants (see below)), the therapist initiated phase three of the evaluation for both the test and control participant. For all participants, sessions were 5 min, with the exception of Kingston who experienced 10 min sessions. Phase three continued for three consecutive sessions. Prior to the start of the session, the therapist placed the alternative response(s) and the control card in a straight line equidistant from the participant. The therapist provided access to the functional reinforcer for 1 min. The session then started upon the presentation of the establishing operation. The therapist placed the alternative, control card, and target responses on extinction. All three sessions in phase three were conducted consecutively with no access to the functional reinforcer between sessions.

#### 2.6.3. Communication Preference Assessment

For participants in the test condition, we also evaluated relative preference for the alternative responses. Once all alternative responses (Alt 1, Alt 2, Alt 3) were mastered, participants then underwent a mixed trial session and communication modality preference assessment prior to proceeding to phase three of the evaluation.

Mixed Trial Session. To minimize a teaching order effect during the communication modality preference assessment, therapists conducted a mixed-trial session for participants assigned to the test condition. During mixed trial sessions, the experimenter placed all three alternative responses and the control card in a straight line equidistant in front of the participant. The trial began with the introduction of the establishing operation and the experimenter prompted the programmed alternative response using a 0 s prompt delay. Three trials of each alternative response were presented within a session (i.e., each session contained 9 total trials). Therapists programmed extinction for the target response, any control card responses, or non-programmed alternative responses (either separately or simultaneously with an alternative response). The therapist also implemented a 3–5 s differential reinforcement of other behavior procedure, placing any alternative responses on extinction if they occurred within 3–5 s of the target or a control card response.
Communication Preference Assessment. For participants assigned to the test condition, we conducted an MSWO communication preference assessment (modified version of Torelli et al., 2015) following a mixed-trial session. During these sessions, the therapist placed all three alternative responses and the control card in a straight line equidistant from the participant. The session began upon the introduction of the establishing operation. Contingent upon any alternative response, the experimenter provided the functional reinforcer for 30 s and simultaneously removed the array. Following 30 s of access to the functional reinforcer, the therapist removed the previously selected alternative response, shifted all alternative responses and control card to the right, and completed this process until either no alternative responses were left to choose from or the participant did not engage in a response within 30 s. The therapist implemented a 3–5 s differential reinforcement of other behavior procedure, placing any alternative responses on extinction if they occurred within 3–5 s of a target or control card response. The therapist did not provide any prompts to engage in an alternative response and, if no selection was made after 30 s, the therapist removed the array briefly and represented the alternative responses and control card.

## 3. Results

### 3.1. Pre-Assessments

Table 2 and Table 3 depict the results of the FA and communication resource interview for all participants who completed all portions of the study, respectively.

### 3.2. Three-Phase Resurgence Preparation

Table 2 depicts the assigned condition and dyad, selected establishing operation, and alternative response phrase and modalities for each participant. Table 4 displays the overall frequency of obtained reinforcers for the alternative response(s) and mean rate of alternative, target, and control card responses during all phases of the preparation. We calculated the mean rate of target responding during phase one of the study with the reinforcement consumption time (Fisher et al., 2019). We specifically calculated mean reinforcement rates in this manner as target responding could occur at any point during a session in an applied setting, including during the 30 s reinforcement period.

#### 3.2.1. Dyad Results

**Figure 1:**
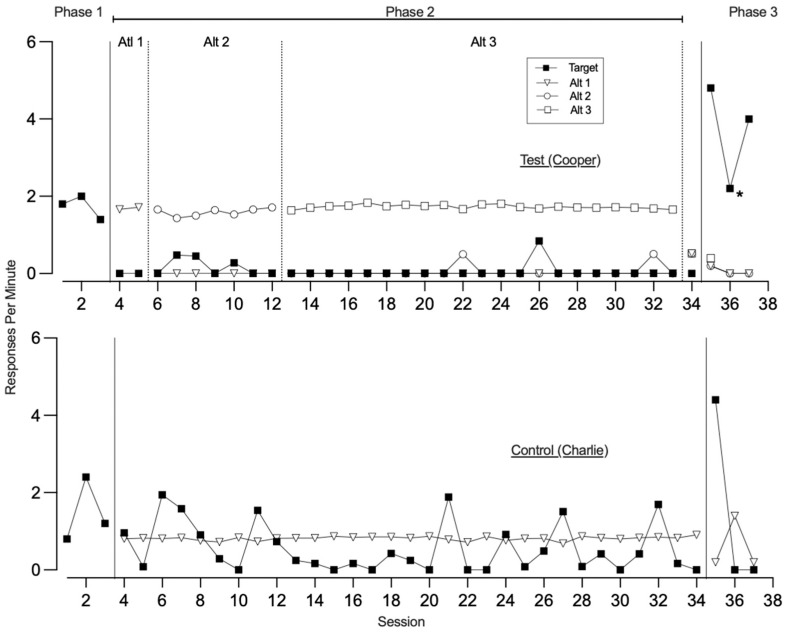
Dyad 1 three-phase resurgence preparation target and alternative responding. Note. Alt is alternative response. Asterisk denotes removal of high-tech alternative response.

**Figure 2:**
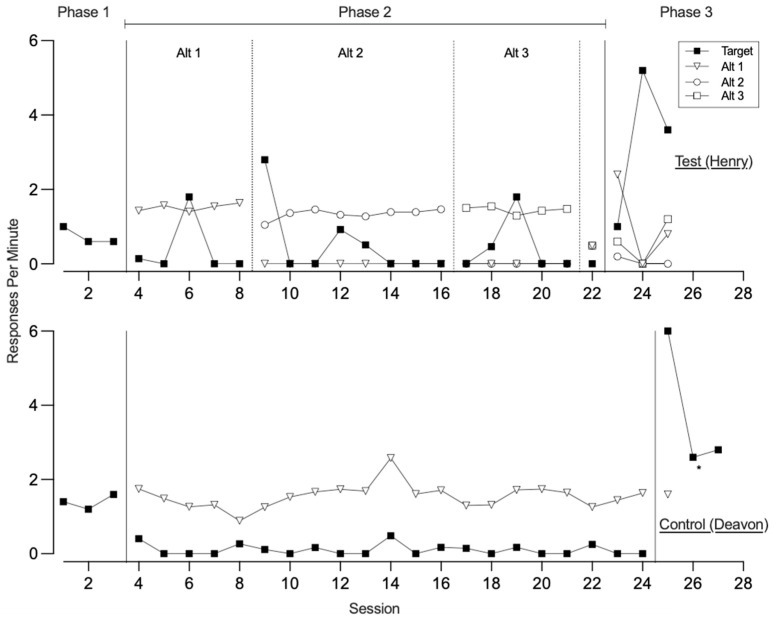
Dyad 2 three-phase resurgence preparation target and alternative responding. Note. Alt is alternative response. Asterisk denotes removal of high-tech alternative response.

**Figure 3:**
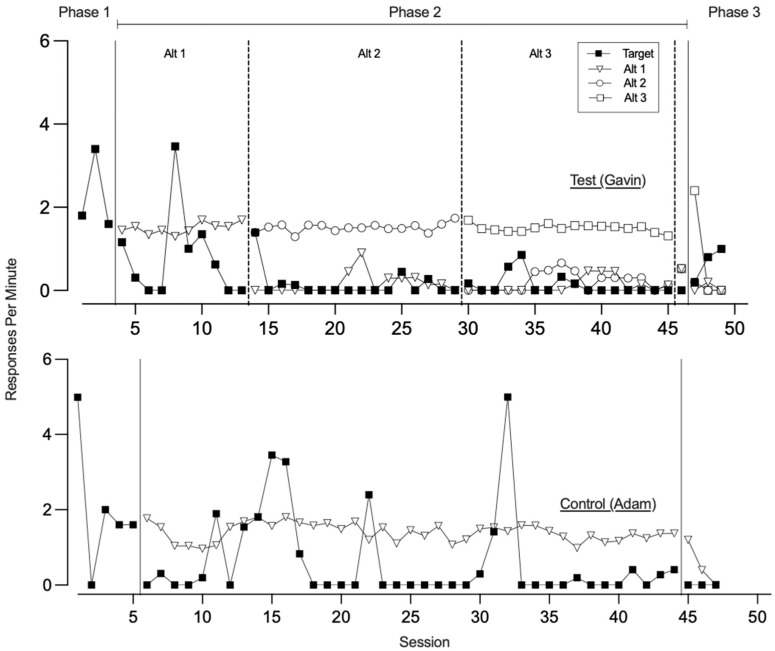
Dyad 3 three-phase resurgence preparation target and alternative responding. Note. Alt is alternative response.

**Figure 4:**
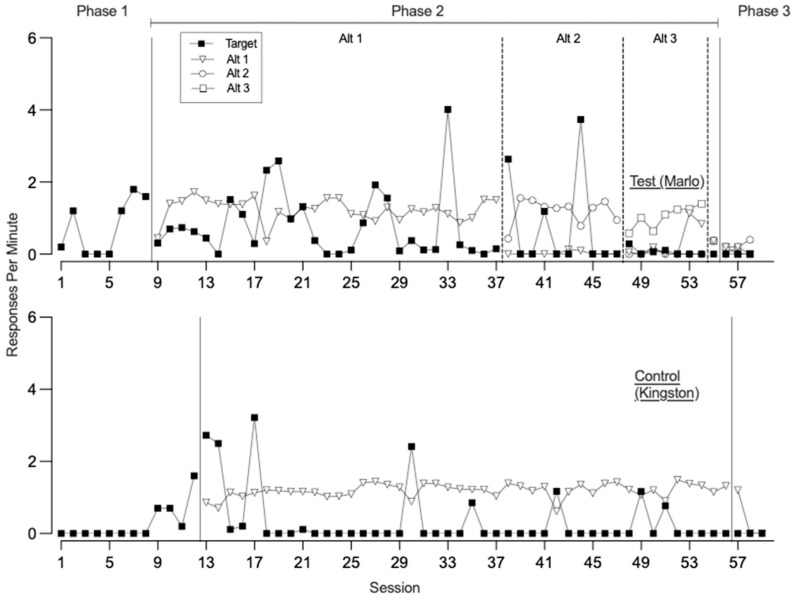
Dyad 4 three-phase resurgence preparation target and alternative responding. Note. Alt is alternative response.

Figure 1, Figure 2, Figure 3 and Figure 4 illustrate the rates of target and alternative responses for individual participants within all dyads, with the top panels representing test condition participants and the bottom panels representing control condition participants.

In phase one, both the test (Cooper) and control (Charlie) condition participants in dyad one (Figure 1) demonstrated similar rates of target responding (*M* = 1.73 and 1.47, respectively), with neither engaging in the control card response. During phase two, the test condition participant was taught to use three alternative responses (Alt 1, Alt 2, and Alt 3) across 2, 7, and 20 sessions, respectively, to access a break and a preferred tangible item. This resulted in consistent suppression of the target response (*M* = 0.07) and minimal engagement with the control card (*M* = 0.03). In contrast, the control condition participant was taught to request a break from blocking access across 30 sessions and showed variable target responding (*M* = 0.55) and low control card responding (*M* = 0.04). In phase three, when all responses were placed on extinction, we observed resurgence for both participants, though their response patterns differed. The test condition participant exhibited high rates of the target response across all three sessions (*M* = 3.67), while alternative responding was low (*M* = 0.26) and primarily occurred in the first session. Control card responding also remained low (*M* = 0.07). Notably, one alternative response (speech-generating device) was discontinued after the first session due to damage, limiting response options in the final two sessions. The control condition participant in phase three displayed high target response rates during the first session, followed by suppression in the remaining two sessions (*M* = 1.47). This participant also showed moderate engagement with the control card (*M* = 0.27) and consistent use of the alternative response across all sessions (*M* = 0.60).

Participants in the second dyad (Figure 2) showed distinct patterns of target responding during phase one. The test condition participant (Henry) exhibited low rates of the target response (*M* = 0.73), whereas the control condition participant (Deavon) demonstrated moderate rates (*M* = 1.40). Both participants engaged equally in control card responses (*M* = 0.07). In phase two, the test condition participant was taught three alternative responses to access preferred items, acquiring Alt 1, Alt 2, and Alt 3 across 5, 8, and 5 sessions, respectively. Throughout this phase, he maintained low rates of the target response (*M* = 0.44) and showed no engagement with the control card. Meanwhile, the control condition participant was taught a single alternative response to request synthesized access to a break, therapist attention, and his preferred item over 20 sessions. This led to a suppression of his target responding (*M* = 0.10), and he did not engage with the control card during this phase. In phase three, both participants demonstrated resurgence of the target response, with elevated and persistent responding across all three sessions (*M* = 3.27 for test condition and 3.80 for control condition participant). Control card responding remained minimal for the test condition participant (*M* = 0.06), while the control condition participant did not engage in the control card response. However, allocation to alternative responses varied between the participants. The test condition participant continued to engage in variable but persistent use of the alternative responses across all sessions (*M* = 0.58). For the control condition participant, researchers removed the alternative response following the first session due to the destruction of the high-tech device. During that initial session, he exhibited a moderate level of alternative responding (*M* = 0.53).

Figure 3 displays the rates of target and alternative responses for dyad 3. In phase one, both the test condition participant and the control condition participant engaged in high rates of the target response (*M* = 2.27 and 2.04, respectively). The test condition participant also showed high rates of control card responding (*M* = 0.93), while the control condition participant engaged in the control card response at a much lower rate (*M* = 0.04). During phase two, the test condition participant was taught three alternative responses to request a break, acquiring Alt 1, Alt 2, and Alt 3 across 10, 15, and 15 sessions, respectively. They demonstrated low rates of the target response (*M* = 0.29) and moderate levels of control card responding (*M* = 0.27). In contrast, the control condition participant was taught a single alternative response to request access to preferred edible items and remained in phase two for 39 sessions. During this time, the control condition participant exhibited variable rates of the target response (*M* = 0.61) and did not engage with the control card. In phase three, when all responses were placed on extinction, resurgence of the target response was observed for the test condition participant but not for the control condition participant. The test condition participant engaged in a low rate of target responding (*M* = 0.67), while the control condition participant did not engage in the target response at all. Both participants displayed similar rates of alternative responding (*M* = 0.31 for the test condition participant and 0.53 for the control condition participant) and engaged with the control card during this phase (*M* = 0.26 and 0.53, respectively).

Figure 4 depicts the response patterns for dyad 4. In phase one, both the test and control condition participants engaged in low to moderate rates of the target response (*M* = 0.75 and 0.27, respectively), with no engagement toward the control card. During phase two, the test condition participant was taught three alternative responses to gain access to the attention of two therapists, acquiring Alt 1, Alt 2, and Alt 3 across 29, 10, and 7 sessions, respectively. Throughout this phase, the test condition participant exhibited variable rates of target responding (*M* = 0.66). Meanwhile, the control condition participant was taught a single alternative response to request a break across 44 sessions and engaged in low to variable rates of the target response (*M* = 0.35). Both participants demonstrated zero or near-zero rates of control card responding (*M* = 0.01 for the test condition participant). In phase three, when all responses were placed on extinction, neither participant engaged in the target response and therefore we did not observe resurgence of the target response. However, their patterns of alternative responding differed. The test condition participant engaged in consistently low levels of alternative responding across all three sessions (*M* = 0.13). In contrast, the control condition participant engaged in high levels of alternative responding during the first session, which then decreased over the second and third sessions (*M* = 0.40). During this phase, the test condition participant also engaged in low rates of control card responding (*M* = 0.13), while the control condition participant did not allocate any responding toward the control card.

#### 3.2.2. Additional Data Analysis

**Figure 5:**
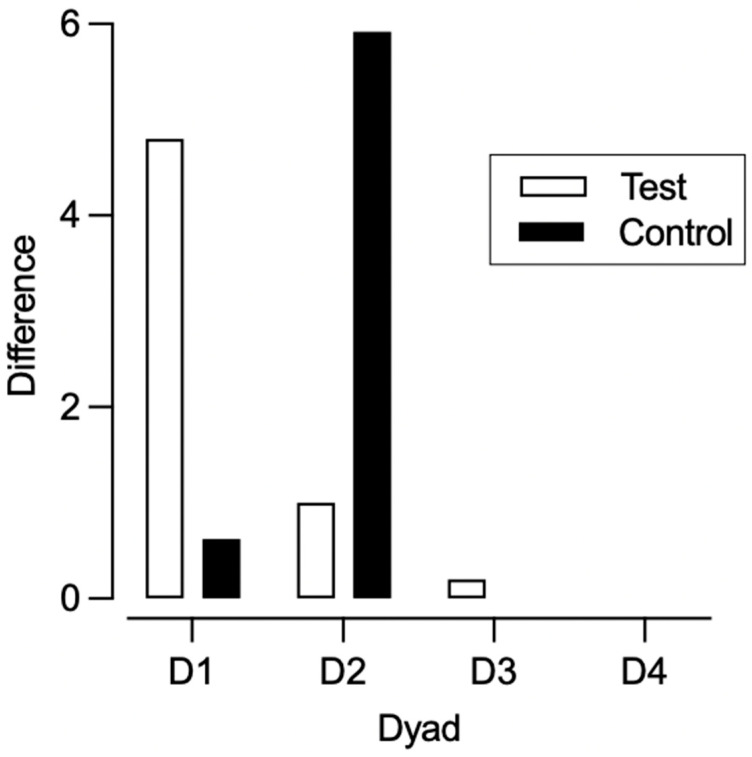
Difference in target responding from phase two to phase three.

Individual Participant Analysis. Overall, we observed resurgence of the target response for five out of eight participants. We observed resurgence for three test condition and two control condition participants (Table 5). Figure 5 depicts the difference between the rate of target responding from phase two to phase three for participants who engaged in target responding during the first session of phase three. In dyad 1, the difference for the control condition participant was 0.62 and 4.80 for the test condition participant. In dyad 2, the difference in target responding was 5.92 for the control condition participant and 1.0 for the test condition participant. For dyad 3, the calculated difference for the test condition participant was 0.2 and the control condition participant did not engage in the target response. Lastly, neither participant in dyad 4 engaged in the target response.

**Figure 6:**
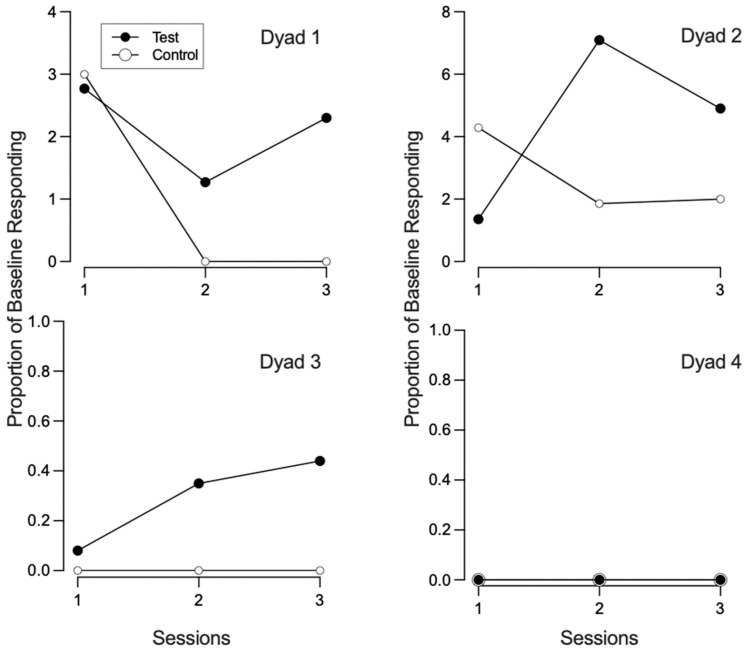
Proportion of baseline across all sessions of phase three.

To account for differences in response rates during phase one and quantify magnitude of resurgence between each dyad, we compared levels of resurgence during phase three to that of phase one for each test and control condition participant, expressed as a proportion of baseline. Figure 6 depicts the proportion of baseline for each session of phase three for both the control and test condition participants within each dyad. In dyad 1, the average proportion of baseline was 2.11 for the test condition participant and 1.0 for the control condition participant. For dyad 2, the proportion of baseline was 4.45 for the test condition participant and 2.72 for the control condition participant. In dyad 3, the proportion of baseline was 0.29 for the test condition participant and 0 for the control condition participant. Finally, in dyad 4, the proportion of baseline was 0 for both the test and control condition participants. When considering all three sessions of phase three, test condition participants exhibited a higher average proportion of baseline in three out of four dyads, while one dyad showed equal average values.

**Figure 7:**
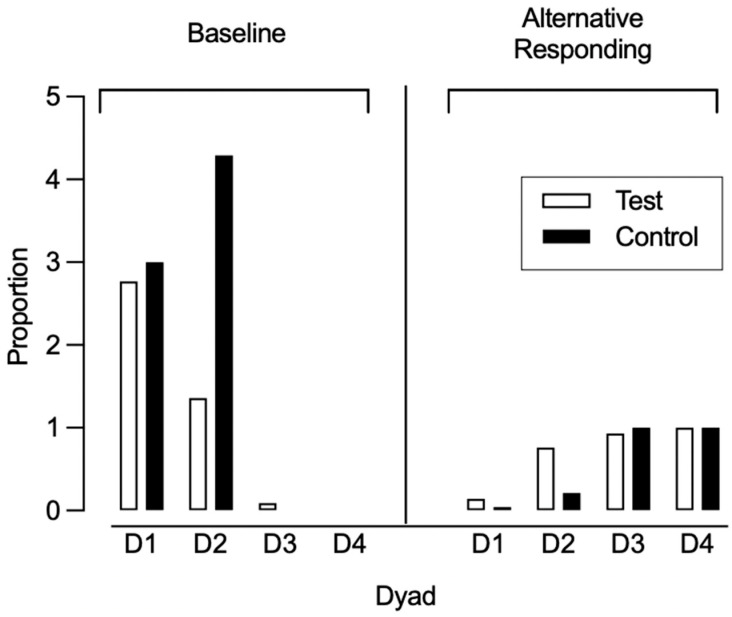
Proportion of baseline and alternative responding for first session of phase three.

Due to one alternative response being removed in phase three for two participants, we also calculated the proportion of baseline only for sessions where all alternative responses remained available across participants. The alterative response was removed after the first session of phase three for both affected participants. Therefore, we compared the proportion of baseline for the first session of phase three across all participants (left half of Figure 7). For dyad 1, the proportion of baseline was 2.77 for the test condition participant and 3.00 for the control condition participant. In dyad 2, the proportion of baseline was 1.36 for the test condition participant and 4.29 for the control condition participant. For dyad 3, the proportion of baseline was 0.08 for the test condition participant and 0 for the control condition participant. Lastly, in dyad 4, both participants had a proportion of baseline of 0. In one out of four dyads, the control condition participants exhibited a higher proportion of baseline (i.e., greater resurgence) in the first session of phase three when compared to the test condition participants, while one dyad exhibited equal values and two dyads similar values.

We also compared the allocation of alternative responding to that of target responding during the first session of phase three for both the test and control condition participants, expressed as a proportion of alternative responding (right half of Figure 7). For dyad 1, the proportion of alternative responding was 0.14 for the test condition participant and 0.04 for the control condition participant. In dyad 2, the proportion of alternative responding was 0.76 for the test condition participant and 0.21 for the control condition participant. In dyad 3, the proportion of alternative responding was 0.93 for the test condition participant and 1.00 for the control condition participant. Lastly, for dyad 4, the proportion of alternative responding was 1.00 for both test and control condition participants. We observed higher responding toward the alternative responses for test condition participants in two out of four dyads.

To evaluate the latency to the first target and alternative response when all responses were placed on extinction during phase three, we recorded the amount of time between the onset of extinction and the occurrence of the first target and alternative response (Table 5). We analyzed the latency to the first target response during the first session of phase three for only participants who engaged in the target response (i.e., two control condition participants and three test condition participants). In dyad 1, the latency to the first target response was 1 s for the control condition participant and 16 s for the test condition participant. The latency to the first alternative response was 3 s for both the control and test condition participant. In dyad 2, the latency to the first target response was 23 s for the control condition participant and 52 s for the test condition participant. The latency for the first alternative response was 51 s for the control condition participant and 4 s for the test condition participant. In dyad 3, the control condition participant did not engage in the target response, whereas the test condition participant experienced a latency of 36 s to the first target response. The latency to the first alternative response was 9 s for the control condition participant and 12 s for the test condition participant. Lastly, in dyad 4, neither participant engaged in the target response, but the latency to the first alternative response was 40 s for the control condition participant and 13 s for test condition participant.

**Figure 8:**
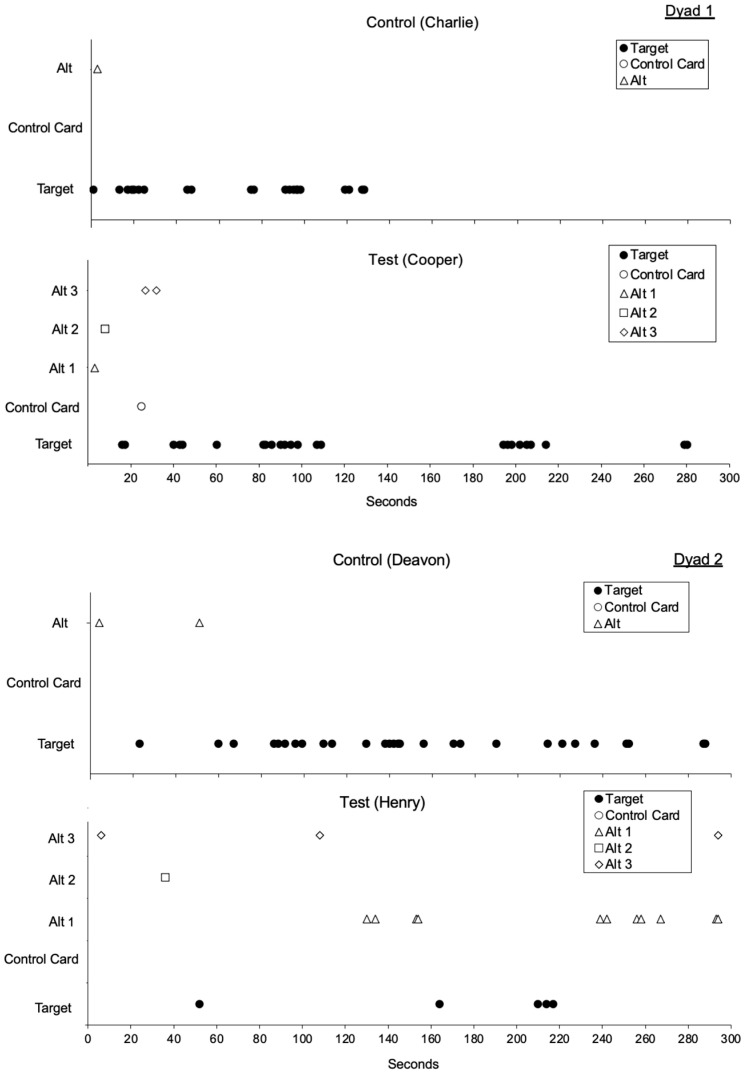
Within-Session Responding for Dyads 1 and 2. Note. Alt is alternative response.

**Figure 9:**
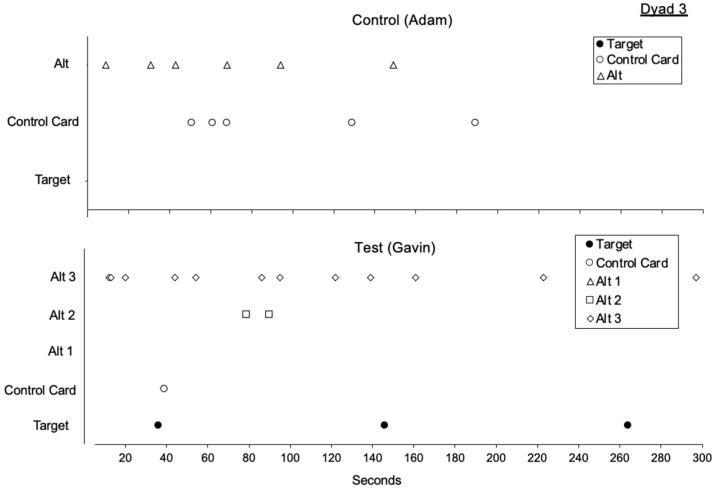

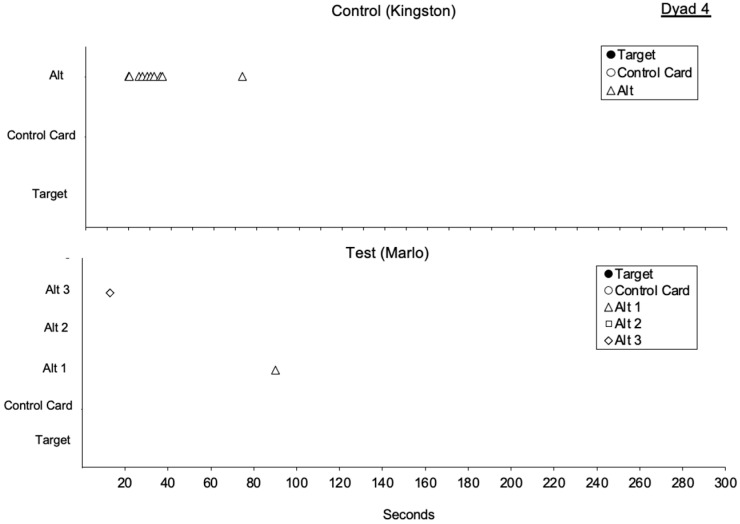
Within-Session Responding for Dyads 3 and 4. Note. Alt is alternative response.

Lastly, we analyzed within-session responding in the first session of phase three (Figure 8 and Figure 9). We did this to identify the first alternative response to emerge for test condition participants and persistence toward the alternative response for each dyad pair. For test condition participants, we observed that the most recently taught modality was the first to emerge during extinction for three out of four test condition participants (Table 6). When comparing within session responding across each dyad, we observed more persistent responding toward the alternative response for test condition participants in three out of four dyads. When taught multiple alternative responses, 25% of test condition participants only engaged in one alternative response prior to the target response emerging, 75% engaged in two alternative responses, and zero participants engaged in all three alternative responses.

**Figure 10:**
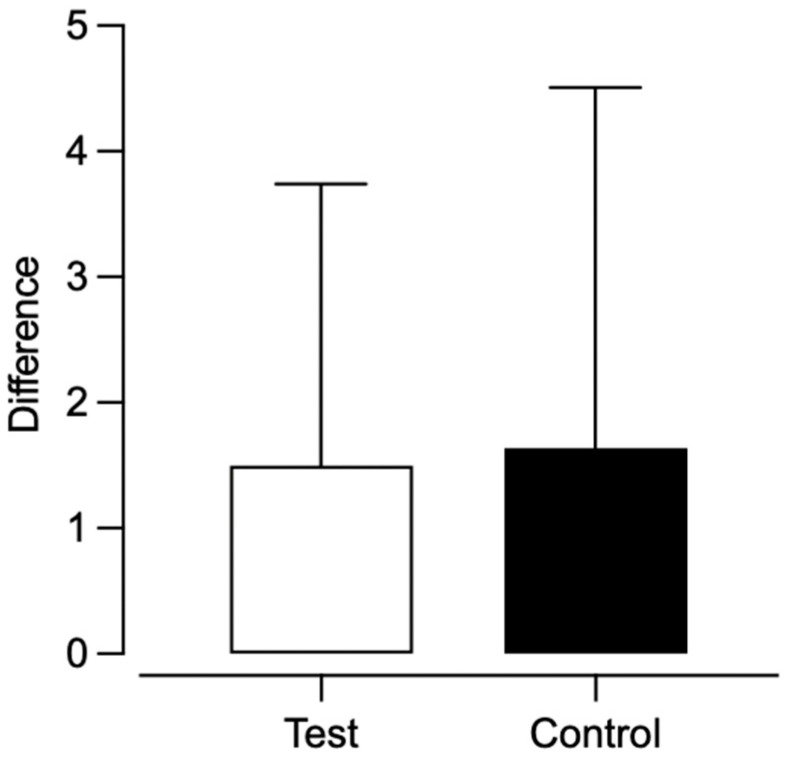
Aggregate difference from phase two to phase three.

**Figure 11:**
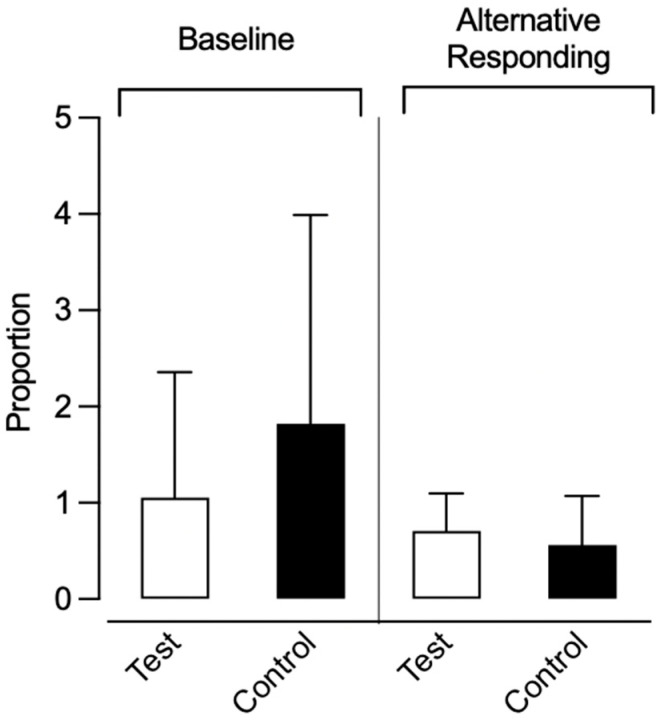
Aggregate test and control condition proportion of baseline and alternative responding for first session of phase three.

Between-Participant Analysis. We also calculated group means and compared them across the test and control conditions. For participants who engaged in target responding during the first session of phase three, the average difference between the rate of target responding from phase two to phase three was 3.27 (range: 0.62–5.92) for control condition participants and 2.00 (range: 0.20–4.80) for test condition participants (Figure 10). This difference was not statistically significant (U = 2.0, *p* = 0.80), though the effect size (Cliff’s Delta = −0.33) suggested a moderate trend favoring the test condition. Figure 11 depicts the proportion of baseline and alternative responding across conditions. Control condition participants experienced a slightly higher proportion of baseline (*M* = 1.82, range: 0–4.29) compared to test condition participants (*M* = 1.05, range: 0–2.77). This difference was not statistically significant (U = 7.0, *p* = 0.88), and the effect size was small (Cliff’s Delta = −0.13), indicating no meaningful difference in proportion of baseline between conditions. There was a minimal difference in proportion of alternative responding between the test (*M* = 0.71, range: 0.14–1.00) and control (M = 0.56, range: 0.04–1.00) condition participants. This difference was not statistically significant (*U* = 8.0, *p* = 1.0), and the effect size (Cliff’s Delta = 0.00) indicated no practical difference between conditions.

When evaluating latency to the first target response, the average latency was 34.6 s for test condition participants and 12.0 s for control condition participants. In summary, when target responding occurred during the first session of phase three, responses in the test condition were, on average, 22.6 s slower under extinction. This difference was not statistically significant (*U* = 5.0, *p* = 0.40), but the effect size (Cliff’s Delta = 0.67) indicated a large effect favoring the test condition. For the alternative response, participants in the test condition engaged in an alternative response within an average of 8 s, compared to 26 s for those in the control condition and an 18 s difference during the first session of phase three. This difference was not statistically significant (*U* = 5.5, *p* = 0.56), though the effect size (Cliff’s Delta = −0.31) indicated a moderate trend favoring the test condition.

#### 3.2.3. Communication Preference Assessment

For all test condition participants, we assessed preference for Alt 1, Alt 2, and Alt 3. A preference hierarchy was identified for all participants. However, the identified most preferred modality for three out of four participants was also the most recently taught alternative response (Table 6). The second and third columns of Table 6 display the communication preference assessment results both before and after phase three. Overall, two out of four participants experienced a change in preference for their most preferred alternative response following contact with extinction during phase three.

## 4. Discussion

Using a between-participants group design and three-phase resurgence preparation, we observed resurgence of the target response for most participants, which is consistent with previous applied literature examining similar questions (e.g., Briggs et al., 2018; Hagopian et al., 1998). Particularly, resurgence of the target response was observed across both test and control condition participants, suggesting that, despite teaching multiple alternative responses, the removal of reinforcement for the alternative response resulted in the return of the previously extinguished target response.

### 4.1. Understanding Impact of Multiple Alternative Responses Across Individuals

There were differences in response patterns across conditions when evaluating responding at the individual level. These differences included the level of target responding from phase two to phase three, proportion of baseline, latency to the first response, and within-session responding. Further analysis of these patterns may shed light on clinical recommendations regarding the impact of teaching multiple alternative responses on resurgence.

To further examine whether training multiple alternative responses affected responding during phase three, we calculated the difference in the rate of target responding from phase two to phase three to numerically quantify the resurgence of target responding that occurred at the individual participant level across dyads. Although resurgence (i.e., observed increase in phase three responding relative to last three sessions of phase two) occurred across both conditions, the results indicated a higher rate of target responding upon contact with extinction for test condition participants in two out of three dyads that engaged in target responding during phase three.

We also evaluated the proportion of baseline to quantify the severity of resurgence when compared to phase one. For the three out of four dyads that engaged in target behavior, we observed that the proportion of baseline across all three sessions in phase three was higher for test condition participants in all dyads when compared to control condition participants, indicating greater resurgence over consecutive sessions of extinction when multiple alternative responses were taught. However, the removal of an alternative response for two participants (e.g., Cooper and Deavon) during the second and third sessions of phase three likely influenced target responding and may have inflated the observed levels of resurgence. To address this, we examined target responding within the first session of phase three separately. On the individual participant level, these results suggested mixed findings, with a higher proportion of baseline for the control condition participants in one dyad and equal or similar proportions of baseline for three dyads. These findings are consistent with previous literature using a similar design, finding no clear difference in the presence, or severity, of resurgence across conditions at the individual level. When considering individual participant findings and comparing across dyads, these results, in addition to previous literature, indicate that teaching multiple alternative responses may not impact the presence or severity of resurgence.

We also observed higher proportions of alternative responding for test condition participants in only two dyads, with similar levels across the remaining dyads. These findings contrast with previous applied studies using within-subject designs (e.g., Lambert et al., 2017) and translational or basic research using between-groups designs (e.g., Fuhrman et al., 2021), which have generally reported greater allocation toward the alternative response. Several methodological differences may help explain our inability to replicate those results. For instance, Lambert et al. (2017) implemented a within-subject design in which participants experienced the control condition prior to the test condition. This sequence may have resulted in repeated exposure to extinction, potentially reducing resurgence and increasing allocation toward the alternative response. Moreover, researchers conducting basic or translational studies, such as Fuhrman et al. (2021) and Nist and Shahan (2023), may exert greater control of reinforcement history for the target response than is typically feasible in applied settings. Such control could influence allocation patterns, leading to higher proportions of alternative responding. Given these differences, future research should continue to explore how teaching multiple alternative responses affects response allocation in applied contexts, while also minimizing repeated exposure to extinction. Based on our current findings, teaching multiple alternative responses may not significantly alter the proportion of alternative responding in the treatment of challenging behavior; however, these results should be interpreted cautiously until they are replicated by other researchers.

Evaluating the latency to the first target and alternative response may also provide clinical insight into the impact of teaching multiple alternative responses. Specifically, even though resurgence may occur, a longer latency to the target response may hold clinical utility, providing caregivers with time to reinforce or prompt an alternative response prior to the target response emerging. In this evaluation, we observed that, when the target response did occur during the first session of phase three, test condition participants exhibited a longer latency to the target response. However, it is important to note that target responding did not occur in two out of four control condition participants and occurred in three out of four test condition participants. Additionally, the difference in average latency across test and control condition participants was only 22.6 s. Although this may present a numerical difference between conditions, these results should be interpreted with caution as 22.6 s may have limited clinical utility and provide caregivers with minimal reaction time prior to the target response occurring. When analyzing the latency to the first alternative response, we observed a similar difference. Although test condition participants were quicker to engage in an alternative response, the difference between latency was only 18 s, again presenting limited clinical utility. These findings suggest that although test condition participants are quicker to respond to the alternative response and slower to engage in the target response, the difference between conditions may have limited impact in the treatment of severe challenging behavior.

Lastly, although the observed differences in latency between test and control conditions were relatively small, within-session data from the first session of phase three revealed greater persistence of alternative responding among test condition participants compared to those in the control condition across most dyads. These findings suggest that, despite minimal differences across other analyses at the individual level (e.g., difference in target responding, proportion of baseline, proportion of alternative responding, and latency), teaching multiple alternative responses may promote overall greater persistence toward alternative responding than teaching a single alternative response. It is important to note that target responding still occurred in three out of four participants in the test condition. Thus, while greater persistence toward the alternative response was observed, the majority of participants still allocated responding to the target behavior. Despite this, the increased persistence of alternative responding may be clinically relevant, especially in situations where communication partners fail to “hear” or “see” an initial alternative response. Furthermore, in a world with increasing use of technology, families are likely to encounter the destruction of a high-tech speech device as a product of challenging behavior, like we encountered in this study. As such, teaching an additional alternative response may provide individuals with additional response options to persist toward if destruction of a device occurs. Interestingly, when evaluating which different alternative responses emerged prior to the target response occurring in phase three, most participants in the test condition engaged in only two of the taught alternative responses, and none engaged in all three. When considering response allocation toward different alternative responses regardless of whether the target response occurred, half of the test participants engaged in all three responses, while the other half engaged in two. These findings suggest that, while teaching multiple alternative responses may enhance persistence, it may not be necessary to teach three different alternative responses.

### 4.2. Understanding Impact of Multiple Alternative Responses Between Groups

In addition to analyzing data at the individual level, we also compared findings between the control and test conditions. Specifically, we compared changes in target and alternative responding between the test and control condition using Mann–Whitney U tests, with Cliff’s Delta reported as a measure of effect size. Among participants who engaged in target responding, the difference in target responding from phase two to phase three was greater, on average, in the control condition than in the test condition. Although this difference was not statistically significant, the effect size suggested a moderate trend favoring the control condition. When examining the proportion of baseline and alternative responding, no statistically significant or practically meaningful differences were observed between the conditions. The control condition showed a slightly higher proportion of baseline responding, while both groups were nearly identical in their proportion of alternative responding. Overall, these findings suggest that teaching multiple alternative responses did not produce reliable group-level differences in either the proportion of baseline or the use of alternative responses during phase three. However, the observed effect sizes indicate trends that may warrant further investigation in studies with larger sample sizes.

Latency to the first target and alternative response during phase three was also compared between conditions. For target responding, participants in the test condition showed longer latencies to engage in the target response compared to those in the control condition, suggesting greater delay to engaging in the target response. Although this difference was not statistically significant, the effect size indicated a large effect favoring the test condition. For alternative responding, test condition participants engaged in alternative responses more quickly than those in the control condition. Again, this difference was not statistically significant, but the effect size suggested a moderate effect favoring the test condition. Together, these latency findings point to potential differences in how quickly participants adjusted to extinction conditions depending on the number of alternative responses trained.

### 4.3. Incorporating Preference for an Alternative Response

Given the potential influence of preference on alternative response persistence and resurgence patterns, we also examined the relationship between alternative response preference, persistence of the alternative response, and resurgence of the target response. Overall, preference for the alternative responses was idiosyncratic and differed across test condition participants. Despite our attempt to control for any influence of teaching order by implementing a mixed trial session prior to the communication modality preference assessment, the highest ranked modality corresponded with the most recently taught modality for three out of four test condition participants. These findings indicate that preference for a communication modality in this evaluation may have been impacted by teaching order. When investigating the relationship between preference and the engagement in the first alternative response during contact with extinction, three out of four individuals engaged in their most preferred modality first, which was also the most previously taught. These findings also suggest that the first response to emerge may have been impacted by the order of teaching or reinforcement, as opposed to mere preference. These findings remain an empirical question and may benefit from additional evaluation. Therefore, future research should investigate the impact that order of teaching has on preference for an alternative response and the role that preference may have on the engagement of an alternative response during contact with extinction.

When evaluating for shift in preference following contact with extinction during phase three, two out of four test condition participants exhibited changes in preference following extinction, suggesting that exposure to extinction contingencies may alter engagement in an alternative response. Interestingly, evident by the results of the communication MSWO preference assessment after phase three, Cooper’s most preferred modality shifted to the alternative response that was removed in the first session and therefore contacted less extinction. These findings add further evidence that contact with extinction may impact the allocation toward an alternative response. Taken together, although results indicated that preference may be influenced by recency of reinforcement, the shift in preference following contact with extinction may suggest positive implications for teaching multiple alternative responses. For instance, there may be times in which a certain alternative response contacts extinction and an individual allocates responding toward other members of the contingency class as opposed to reallocation to the target response. Therefore, providing multiple alternative responses may provide individuals with other response options, particularly when certain modalities may contact extinction within the natural environment.

### 4.4. Summary

The results of this evaluation differ when evaluating at the individual and group levels. Similar to previous literature, observed results at the individual level suggest that teaching multiple alternative responses is unlikely to prevent the presence, or severity, of resurgence of the target response. Given these findings, we are hesitant to recommend this approach as a strategy for mitigating resurgence. Contrary to previous literature, our results indicated a minimal difference in the proportion of responding toward the alternative response. Given that our findings contradict prior literature, future research should continue to investigate the impact that teaching multiple responses has on the allocation toward the alternative response.

At the group level, results indicate that teaching multiple alternative responses may lead to reduced resurgence, less severe resurgence, and minimal differences in alternative responding. However, all group-level comparisons were statistically non-significant, and effect sizes, while suggestive of trends, were generally small to moderate. These findings should be interpreted with caution, particularly given the small sample size. Future research using between-group designs and larger samples is needed to reduce sampling bias and improve the generalizability of these findings.

In addition to the minimal effect on resurgence, our evaluation also provides limited insight into the role of preference for an alternative response. Given our inability to differentiate preference from teaching order, future research should continue to investigate the impact that preference has on both the alternative and target response upon contact with extinction.

Despite these findings, there may be situations in which clinicians may still opt to teach multiple alternative responses. First, our findings indicate that teaching multiple alternative responses may enhance the persistence of alternative behavior. This may be particularly useful for individuals using a high-tech communication device, as it may be beneficial to teach one additional low-tech alternative response to maintain communication when the device is unavailable or damaged. Secondly, our findings also indicated that allocation toward an alternative response may be impacted following contact with extinction. Therefore, teaching multiple alternative responses may provide the individual with other responses to allocate toward if certain alternative responses contact extinction in the natural environment. Although teaching multiple alternative responses may be beneficial for these reasons, clinicians should carefully weigh the potential benefits and drawbacks of teaching multiple alternative responses. This includes considering the feasibility of implementation—such as the time and resources required in both clinical and home settings—and prioritizing clinical goals. For instance, it may be more effective to first focus on establishing a single alternative response, achieving quicker suppression of the target response, and progressing through schedule thinning before teaching an additional response(s).

This study addressed several gaps and limitations of previous literature and therefore this evaluation offers several strengths that can guide future research in this area. Notably, we employed a between-participants group design and minimized exposure to extinction. Although balancing the number of reinforcers for the alternative response posed some challenges, future research should continue to investigate the use of similar designs as an alternative to within-subject replication when evaluating the impact that variables may have on resurgence. Second, we incorporated a control response to ensure that responding was under the control of reinforcement and not extinction-induced variability (see Lattal & Oliver, 2020, for discussion). As such, when participants engaged in the target response, the rate of target responding consistently exceeded that of the control card response, suggesting that these instances were more likely due to resurgence rather than extinction-induced variability. For participants in the test condition, the rate of alternative responding was consistently higher than that of the control card, further supporting the interpretation that allocation toward the alternative responses was maintained by reinforcement. It is important to note that one test condition participant engaged in moderate rates of the control card response during phase three; however, they engaged in similar rates across all phases of the evaluation. Lastly, one control condition participant engaged in a higher rate of control card responses when compared to alternative responses in phase three; however, this was most likely due to his high rates of stereotypic finger tapping. Future research should continue to incorporate a control response to ensure that responding is under the control of reinforcement. When incorporating a control response, future research should also ensure that the control card is topographically dissimilar from the other response options. Particularly, we used a black laminated card as the control response, which may have shared physical properties with other alternative responses (e.g., card touch, card exchange). This similarity could have influenced response allocation, making it difficult to rule out response generalization as a contributing factor.

This study presents additional strengths, such as programming only externally aided communication modalities (e.g., speech-generating device, card touch, card exchange) to ensure consistent use of physical stimuli, limiting confounding variables that may have impacted responding across multiple alternative responses. Despite this change in procedures when compared to other applied research (Lambert et al., 2017), we still observed less responding toward the alternative response when compared to the target response and future research should continue to investigate this. Lastly, we programmed topographically dissimilar alternative and target responses, ensuring that responding was not a result of response generalization due to similarity in response topographies.

### 4.5. Limitations and Future Research

This study also had limitations, presenting areas of improvement and investigation for future research. Heterogeneous response patterns within a small sample may have obscured the ability to detect reliable group differences, despite effect sizes suggesting possible trends. Future studies with larger samples are needed to better characterize these patterns and clarify whether group-level effects emerge under more highly powered conditions. We offer the following points that may have contributed to the heterogeneity of outcomes. First, we did not implement a variable interval (VI) schedule of reinforcement for the target response during phase one, resulting in unequal reinforcement rates across conditions. A continuous reinforcement schedule was used instead to reflect typical clinical practice during baseline procedures. Additionally, we balanced obtained reinforcers for the alternative response differently when compared to previous research. Rather than using a fixed session duration in phase two, we employed trial-based sessions, which introduced variability in session lengths across participants and dyads. Although the total number of reinforcers was matched within 10% across conditions, reinforcement rates were equivalent in only three of the four dyads. Reinforcement was also not evenly distributed across the specific alternative responses (Alt 1, Alt 2, Alt 3) in the test condition. Future research should consider the use of a VI schedule and fixed duration sessions to ensure the balancing of reinforcement rates across conditions for the alternative and target responses. Second, participants were not matched into dyads based on participant characteristics. The contingencies maintaining some participants’ target responding differed across dyad pairs. Specifically, in dyads 2 and 4, one participant’s target responding was maintained by an isolated contingency while the other participant’s target responding was maintained by a synthesized contingency. This difference in reinforcement quality could have impacted the levels of target and alternative responding in phase three. Additionally, participants within dyads may have experienced different language levels. Future research may consider matching participants into dyads based on similar participant characteristics to allow for better comparison across each dyad.

Contrary to previous research procedures, we chose to block non-targeted alternative responses during phase two to limit exposure to extinction, which may have contributed to reduced allocation toward those responses in phase three. However, we would expect that response blocking, or extinction, would result in decreased allocation toward the alternative response in phase three, which was not observed and therefore it is possible that this did not affect alternative responding. Nonetheless, future research should consider the use of extinction versus response blocking for the non-targeted alternative response when teaching multiple alternative responses.

Notably, two participants destroyed their high-tech communication devices, necessitating the removal of that modality after session one of phase three. As a result, not all acquired response topographies were available throughout the entire phase, limiting the conclusions that can be drawn from sessions two and three. Given the widespread use of high-tech devices in communication, future research should explore how response restriction (e.g., due to device destruction or unavailability) impacts both the resurgence of target behavior and the persistence of alternative responses (Hagopian et al., 2011). An additional limitation was the variability in target responding during phase two across conditions, which reflects target responding in an applied setting. Further research is needed to examine how exposure to extinction for the target response in phase two may impact resurgence.

Finally, while previous studies have identified strategies for mitigating resurgence—such as the use of discriminative stimuli and reinforcement schedule manipulations—this study did not incorporate discriminative stimuli and instead used dense reinforcement schedules for alternative responses. In clinical practice, it is rare for FCT to be implemented and terminated using dense schedules without schedule thinning. It is therefore possible that a combination of multiple alternative responses, reinforcement schedule thinning, and discriminative stimuli (e.g., multiple or chained schedules; Hanley et al., 2001) may more effectively mitigate resurgence. Future research should evaluate such combined strategies.

## Figures and Tables

**Table 1:** Participant demographics.

Participant	Age	Sex Assigned at Birth	Race	Diagnosis	Spoken Language(s)
Henry	7	Male	Asian American	ASD	English, Spanish
Charlie	9	Male	White	ASD	English
Cooper	10	Male	White	ASD, ODD, ADHD	English
Deavon	15	Male	White	ASD, ID	English
Gavin	16	Male	Hispanic	ASD, ADHD	English
Marlo	13	Female	Hispanic	ASD, ID, language impairment, other specified stereotypic movement disorder	English, Spanish
Kingston	16	Male	Black	Other specified disruptive, impulse control, and conduct disorder; Down syndrome	English
Adam	8	Male	Hispanic	ASD, specified stereotypic movement disorder, other specified disruptive, impulse control, and conduct disorder	English, Spanish

Note. ASD = Autism Spectrum Disorder; ID = Intellectual Disability; ADHD = Attention Deficit Hyperactivity Disorder; ODD = oppositional defiant disorder.

**Table 2:** Dyad assignment and resurgence preparation features.

Participant Dyad	Target Responses	Identified Function(s) and Selected Establishing Operation	Alternative Response Modalities	Alternative Response Phrase
Dyad 1				
Control	Aggression, disruption, self-injury	**Escape from blocking access**	SGD	“My Way”
(Charlie)				
Test	Vocal protests, aggression, self-injury, disruption	Escape, **tangible**	Card touch, card exchange, SGD	“Toys Please”
(Cooper)				
Dyad 2				
Control	Aggression, disruption, self-injury, elopement	**Synthesized escape to attention and tangible**	SGD	“Hang Out”
(Deavon)				
Test	Aggression, disruption, self-injury	Escape, **tangible**, attention	Button, card exchange, SGD	“Toys please”
(Henry)				
Dyad 3				
Control	Aggression, disruption, self-injury	**Tangible (edible)**	Card exchange	“Snack”
(Adam)				
Test	Aggression, self-injury	**Escape**, attention, tangible	SGD, card touch, card exchange	“Hang out”
(Gavin)				
Dyad 4				
Control	Aggression, disruption	**Synthesized escape to tangible**	Card touch	“Break”
(Kingston)				
Test	Aggression, disruption, self-injury	**Diverted attention**	Card touch, SGD, card exchange	“Hang out please”
(Marlo)				

Note. Bolded function is selected establishing operation for three-phase resurgence preparation. SGD is speech-generating device.

**Table 3:** Communication resource interview weighted scores.

Modality	Weighted Scores Across Participants							
	Control Condition				Test Condition			
	Charlie	Deavon	Kingston	Adam	Henry	Cooper	Gavin	Marlo
SGD	**2**	**2**	3	5	**4**	**6**	**2**	**2**
Card Exchange	4	6	8	**2**	**4**	**2**	**6**	**6**
Card Touch	8	4	**3**	5	8	**4**	**4**	**4**
Button	6	8	6	8	**4**	8	8	8

Note. Taught alternative response modalities are bolded. SGD is speech-generating device.

**Table 4:** Mean rate for target, alternative, and control card responses.

	Phase One		Phase Two			Phase Three		
Name	TR	Control	TR	Alt	Control	TR	Alt	Control
Dyad 1								
Control (Charlie)	1.47	0	0.55	0.81 (319)	0.04	1.47	0.60	0.27
Test (Cooper)	1.73	0	0.07	1.72 (320)	0.03	3.67	0.27	0.07
Dyad 2								
Control (Deavon)	1.40	0.07	0.10	1.55 (200)	0	3.80	0.53	0
Test (Henry)	0.73	0.07	0.45	1.42 (195)	0	3.27	0.87	0.06
Dyad 3								
Control (Adam)	2.04	0.04	0.61	1.40 (390)	0	0	0.47	0.53
Test (Gavin)	2.27	0.93	0.28	1.67 (435)	0.24	0.67	0.93	0.26
Dyad 4								
Control (Kingston)	0.27	0	0.35	1.19 (440)	0	0	0.40	0
Test (Marlo)	0.75	0	0.67	1.24 (482)	0.01	0	0.40	0.13

Note. Total number of obtained reinforcers is in parentheses. Alt is alternative response. TR is target response.

**Table 5:** Presence of resurgence and latency to target and alternative responses.

Dyad (Name)	Resurgence (+/−)	Alternative Response Latency (s)	Target Response Latency (s)
Dyad 1			
Control (Charlie)	+	3	1
Test (Cooper)	+	3	16
Dyad 2			
Control (Deavon)	+	51	23
Test (Henry)	+	4	52
Dyad 3			
Control (Adam)	−	9	N/A
Test (Gavin)	+	12	36
Dyad 4			
Control (Kingston)	−	40	N/A
Test (Marlo)	−	13	N/A

Note. Plus signs indicate the presence of resurgence. Minus signs indicate the absence of resurgence. N/A is not applicable.

**Table 6:** Communication modality preference results.

Name	Most Preferred Alternative Response Before Phase Three	Most Preferred Alternative Response After Phase Three	First Response to Emerge Phase Three
Henry	SGD (alt 3)	SGD (alt 3)	SGD (alt 3)
Cooper	Card exchange (alt 2)	SGD (alt 3)	Card touch (alt 1)
Gavin	Card exchange (alt 3)	Card exchange (alt 3)	Card exchange (alt 3)
Marlo	Card exchange (alt 3)	SGD (alt 2)	Card exchange (alt 3)

Note. SGD is speech-generating device.

## Data Availability

All accessible data are contained in the manuscript.

## Supplementary Materials

The following supporting information can be downloaded at: https://www.mdpi.com/article/10.3390/bs15081014/s1, Communication Resource Interview.

## Abbreviations

The following abbreviations are used in this manuscript:

FA	Functional analysis
Alt	Alternative response
FCT	Functional communication training
MSWO	Multiple stimulus without replacement
