# Supplementary material for: The Impact of Teaching Multiple Responses on Resurgence of Target Behavior and Persistence of Alternative Responding
Source: Behav Sci (Basel). 2025 Jul 25;15(8):1014. doi: 10.3390/bs15081014 (PMC12382980; doi:10.3390/bs15081014)
Supplement: Supplementary file 1 [file behavsci-15-01014-s001.zip › behavsci-3649722-supplementary.pdf]

Communication Resource Interview

This form includes questions to gauge your child's current communication skills, history with prior communication modalities, along with your preference for certain communication modalities and perceived feasibility.

Please fill out questions below. If you are unsure what a certain communication modality is, please ask the clinician to provide further information and a demonstration.

This form was informed by the work of Gibson et al. (2020) and modified to include additional details to better assist with identifying communication preference.

1. Child's Name: ______________________

2. Please describe your child's current visual functioning:

   a. Vision is within normal limits
   b. Corrected vision impairment (e.g., glasses)
   c. Cortical visual impairment
   d. Uncorrected vision impairment
   e. Unknown

3. Please describe your child's current hearing status:

   a. Hearing is within normal limits
   b. Corrected hearing impairment (e.g., hearing aids, cochlear implant)
   c. Uncorrected hearing impairment
   d. Unknown

4. How does your child currently let you know what they want (for example, food, toys, or attention)? Please check ***all*** that apply

   Leads or brings items to others
   Cries or engages in challenging behavior
   Points to items
   Signs or uses sign language
   Exchanges or uses pictures or symbols
   Uses a speech output device or iPad
   Engages in sounds or babbling
   Uses single words
   Uses sentences
   Other (please describe)

   ____

5. Has your child EVER used any of the following to communicate (even if they are not using it now)?

   Leads or brings items to others
   Cries or engages in challenging behavior
   Points to items
   Signs or uses sign language
   Exchanges or uses pictures or symbols
   Uses a speech output device or iPad

Engages in sounds or babbling
Uses single words
Uses sentences
Other (please describe)

____

6. If your child uses signs, which ones do they use most often? (place N/A if not applicable)

____

____

7. If your child uses pictures or symbols, which ones do they use most often? (place N/A if not applicable)

____

____

8. If your child uses an iPad or speech output device, what do they ask for most often? (place N/A if not applicable)

____

____

9. If your child uses spoken words, how many words to they use without assistance?

   a. 1–3
   b. 4–6
   c. 7–10
   d. More than 10
   e. Does not use single words independently

10. If your child uses a speech output device or iPad, how many items can they request without assistance?

   a. 1–3
   b. 4–6
   c. 7–10
   d. More than 10
   e. Does not use device or application independently

11. If your child asks for things with spoken words, please indicate what they ask for most often: (place N/A if not applicable)

____

12. Please rank the following communication modalities in order that ***you*** would like your child to use. 1 being the most liked, and 4 being the least liked.

	1	2	3	4
Picture card exchange	○	○	○	○

Picture card touch	○	○	○	○
iPad speech device	○	○	○	○
Speech generating button	○	○	○	○

13. Please share any information as to why you selected communication modalities in the order that you did (why do you prefer the top communication modality over the others)?

____

____

14. Please rank the following communication modalities in order that ***<u>you think your child would like</u>*** to use. 1 being the most liked, and 4 being the least liked.

	1	2	3	4
Picture card exchange	○	○	○	○
Picture card touch	○	○	○	○
iPad speech device	○	○	○	○
Speech generating button	○	○	○	○

15. Please rank the following communication systems in order of easiest to implement with your child in any setting. 1 being the easiest to implement, and 4 being the hardest to implement.

	1	2	3	4
Picture card exchange	○	○	○	○
Picture card touch	○	○	○	○
iPad speech device	○	○	○	○
Speech generating button	○	○	○	○

16. Please indicate if your family currently has access to the items listed below. The approximate cost of each item is listed below:

	Yes, I have access to this item.	No, I do not have access to this item, BUT would be able to purchase the items	No, I do not have access to this item and would NOT be able to able to purchase it.
iPad for communication only (~$350)	○	○	○
Communication application for iPad (~$50–$300)	○	○	○
Printer and ink (~$150 if purchasing own)	○	○	○
Laminator and supplied (~$50)	○	○	○
Velcro (~$20)	○	○	○
